# Mothers’ knowledge, attitude, and behavior regarding child immunization, and the association with child immunization status in Medan City during the COVID-19 pandemic

**DOI:** 10.1016/j.ijregi.2023.03.014

**Published:** 2023-05-04

**Authors:** Bryant Elbert, Cut Meliza Zainumi, Raden Ajeng Dwi Pujiastuti, Muhammad Rizki Yaznil, Gema Nazri Yanni, Ivana Alona, Inke Nadia D. Lubis

**Affiliations:** a Faculty of Medicine, Universitas Sumatera Utara, Medan, Indonesia; b Department of Anesthesiology and Intensive Care, Faculty of Medicine, Universitas Sumatera Utara, Medan, Indonesia; c Department of Neurology, Faculty of Medicine, Universitas Sumatera Utara, Medan, Indonesia; d Department of Obstetrics and Gynecology, Faculty of Medicine, Universitas Sumatera Utara, Medan, Indonesia; e Department of Community Medicine, Faculty of Medicine, Universitas Sumatera Utara, Medan, Indonesia; f Department of Pediatrics, Faculty of Medicine, Universitas Sumatera Utara, Medan, Indonesia; g Menzies School of Health Research, Darwin, Australia

**Keywords:** Immunization, Knowledge, Attitude, Behavior, Children, COVID-19

## Abstract

**Background:**

The COVID-19 pandemic has caused disruption to healthcare access worldwide and has impacted basic childhood immunization services. A decline in immunization coverage can cause immunity gaps and lead to outbreaks of vaccine-preventable diseases. Our study evaluated the association between mothers’ knowledge, attitude, and behavior regarding immunization and child immunization status during the COVID-19 pandemic in Medan, Indonesia.

**Methods:**

An analytical cross-sectional study was conducted from April to November 2021. Mothers with children aged 0–12 months were interviewed about their knowledge, attitude, and behavior regarding immunization, and their child's immunization status.

**Results:**

Of 196 participants, 46.5% had low knowledge on immunization, 41.3% had a negative attitude, and 20.4% had negative behavior. Only 62.8% of participants had children with a complete vaccination status, and mothers with moderate knowledge (OR 2.65, 95% CI 1.08–6.61), negative attitude (OR 5.33, 95% CI 2.71–10.59), and negative behavior (OR 7.88, 95% CI 3.36–19.47) were more likely to not vaccinate their children.

**Conclusion:**

Mothers’ attitude, behavior, and educational background were associated with child immunization status. Recovery efforts to improve immunization coverage are urgently needed, and should include efforts to reduce mothers’ hesitancy regarding child vaccination during the pandemic.

## Highlights

• Disruption to healthcare access due to the COVID-19 pandemic impacted child immunization
• Children of mothers with a higher education had a higher immunization rate
• Mothers with a lower education were more likely to have low knowledge on immunization
• Most mothers hesitated to vaccinate their children during the COVID-19 pandemic
• Mothers with negative attitude and behavior would prefer to not vaccinate their children

## Introduction

The COVID-19 pandemic caused an interruption to healthcare access worldwide. Indirect impacts included a disruption to childhood immunization services in at least 85 countries. Routine childhood immunization is fundamental for the health of children. Through immunization, the spread of diseases (e.g. diphtheria, pertussis, tetanus, hepatitis B, polio, and measles) and deaths in children under five can be prevented. However, due to the COVID-19 pandemic, global immunization coverage dropped from 86% in 2019 to 83% in 2020. An estimated 23 million children under the age of 1 year did not receive their third dose of diphteria-tetanus-pertussis (DTP) vaccine, an increase of 3.7 million from 2019. In the WHO South-East Asia region, the drop in immunization coverage was estimated at 57% [1].

The magnitude of disruption to health systems was greater in low- and middle-income countries, with 60% of children under 1 year old who missed immunization being from Angola, Brazil, Democratic Republic of the Congo, Ethiopia, India, Indonesia, Mexico, Nigeria, Pakistan, and the Philippines [1,2]. In Indonesia, childhood immunization coverage dropped from 93.7% in 2019 to 82.6% in 2020, and remained at 84.2% in 2021 [3]. Similarly, in North Sumatera province, a decrease from 86.2% in 2019 to 75.5% in 2020 was reported [4].

The causes of disruption to routine immunization included problems related to healthcare workers, insufficient supply of personal protective equipment, travel restrictions, social distancing measures, disrupted vaccine supply chain, and fear among people [5]. Parents’ knowledge, attitude, and practices regarding immunization are also contributing factors in making decisions about their children's immunization [6].

Our study evaluated the association between mothers’ knowledge, attitude, and behavior regarding immunization and child immunization status in North Sumatera province, Indonesia during the COVID-19 pandemic.

## Methods

A cross-sectional study was conducted among mothers of children aged 0–12 months in Medan City, from April to November 2021. The inclusion criteria were mothers of children aged 0–12 months old who previously gave birth at Universitas Sumatera Utara Hospital, Medan, between September 2020 and September 2021. The mothers were randomly selected from the hospital list, and were contacted via WhatsApp. The study information was provided, and informed consent was obtained from all participants. Mothers were then asked to complete an online questionnaire about their knowledge, attitudes, and practices towards their children's basic immunization, which was adapted from other studies [7,8]. The questionnaire was adapted for the COVID-19 situation, and included questions on sociodemographic characteristics, knowledge, attitude, behavior, vaccination status, child's age, and number of children. Knowledge and behavior were scored 1 for every correct choice, while the attitude choices were classified into strongly disagree, disagree, neutral, agree, and strongly agree, which were scored 1 to 5, respectively.

All data was analyzed using SPSS software, version 26. Median and range were calculated for continuous variables and frequencies and percentages for categorical variables. Continuous variables were then converted into categorical variables. Normal distribution was tested using the Kolmogorov–Smirnov test. Where data were normally distributed, the analysis was parametric, while data that were not normally distributed underwent non-parametric analysis using chi-squared. Multiple correlation analysis was used to assess correlation between knowledge, attitude, and behavior regarding child immunization coverage. Level of knowledge was categorized into three groups: low, moderate, and high. Data giving scores of 0–4, 5–6, and 7–8 were considered low, moderate, and high, respectively. Attitude and behavior were categorized into two groups: positive and negative. Data scoring below the median were considered negative, and the rest were considered positive.

The study was approved by the Research Ethics Committee of the Faculty of Medicine, Universitas Sumatera Utara (no. 846/KEP/USU).

## Results

In total, 344 mothers met the inclusion criteria and were messaged via WhatsApp. Of these, 196 (56.9%) agreed to participate and were enrolled in the study. The median age of the mothers was 31.5 years, with range of 24–46 years, 50.5% of the mothers were housewives, 64.3% had a bachelor degree, and 59.7% were mothers with two children (Table 1).

**Table 1:** Mothers’ sociodemographic characteristics.

Characteristics	Frequency *n* (%)
Mother's age	
24–29	75 (38.3)
30–39	111 (56.6)
40–46	10 (5.1)
Occupation	
Housewife	99 (50.5)
Employee	40 (20.4
Medical staff	23 (11.7)
Entrepreneur	16 (8.2)
Other (teacher, civil servant, priest, insurance agent)	18 (9.2)
Mother's dducation	
Bachelor degree	126 (64.3)
High school	60 (30.6)
Middle school	6 (3.1)
Elementary school	4 (2.0)
Number of children	
1	53 (27.0)
2	117 (59.7)
3–4	26 (13.3)

The analysis of vaccination knowledge showed that the majority of participants (87.8%) understood that if a vaccine dose was missed, they should visit a medical facility and seek advice, 74% knew that immunization did not cause autism, and 68.9% participants knew that immunization did not cause impotency. However, only a little over half of participants (55.1%) knew that if a vaccine dose was missed, the schedule did not need to be restarted, 51.5% knew that immunization did not prevent non-communicable diseases, 51% knew that immunization did not contribute to a child's brain development, 50.5% knew that vaccines do not contain nutritional supplements, and fewer than half of the participants (45.4%) knew that a vaccine was not a growth factor (Supplementary Table 1).

Of 196 participants, 123 (62.8%) had completed their child's immunization, while 143 (72.9%) had low–moderate knowledge regarding immunization. Mothers with a high education level (OR 12.5, 95% CI 3.71–42.1, *p* < 0.001) were more likely to have high knowledge regarding immunization than mothers with a moderate education level (high school graduate), while mothers with two children (OR 2.60, 95% CI 1.11–6.06, *p* = 0.02) were more likely to have higher knowledge on immunization than mothers with only one child (Table 2).

**Table 2:** Association between mothers’ demographic characteristics and knowledge regarding immunization.

Characteristics	Knowledge		Total	Odds ratio (95% CI)*	*p*-value
	High (%) *n* = 53	Low–moderate (%) *n* = 143			
Mothers’ age					
24–29	17 (22.7)	58 (77.3)	75	Ref	
30–39	32 (28.8)	79 (71.2)	111	1.38 (0.70–2.72)	0.35
40–46	4 (40.0)	6 (60.0)	10	2.27 (0.57–9.00)	0.24
Occupation					
Housewife	24 (24.2)	75 (75.8)	99	Ref	
Employee	8 (20.0)	32 (80.0)	40	0.78 (0.31–1.92)	0.59
Medical staff	9 (39.1)	14 (60.9)	23	2.00 (0.77–5.22)	0.15
Entrepreneur	5 (31.3)	11 (68.8)	16	1.42 (0.44–4.49)	0.55
Others	7 (38.9)	11 (61.1)	18	1.98 (0.69–5.70)	0.20
Mothers’ education					
Bachelor degree	50 (39.7)	76 (60.3)	126	12.5 (3.71–42.11)	< 0.01
High school	3 (5.0)	57 (95.0)	60	Ref	
Elementary–middle school	0 (0.0)	10 (100.0)	10	0.08 (0.04–16.28)	0.087
Number of children					
1	8 (15.1)	45 (84.9)	53	Ref	
2	37 (31.6)	80 (68.4)	117	2.60 (1.11–6.06)	0.02
3–4	8 (30.8)	18 (69.2)	26	2.50 (0.81–7.67)	0.10

⁎ Logistic regression

Analysis on attitudes revealed that the majority of participants (81.7%) agreed that child immunization during the COVID-19 pandemic was important, 76.1% agreed that vaccination provides more benefits than harm, 74.5% disagreed that healthy children do not need to be vaccinated. However, there remained 34.7% of participants who felt neutral towards the statement that compliance with vaccination schedules during the COVID-19 pandemic was important, while 30.1% participants also felt neutral regarding the idea that in the case of vaccine-preventable disease (VPD) emergence, non-vaccinated children should be quarantined (Supplementary Table 2). Based on these findings, the mothers in this study were observed to have equally positive (53.6%) and negative (46.4%) attitudes, although mothers who were employees and high school graduates were less likely to have positive attitude (Table 3).

**Table 3:** Association between demographic characteristics and attitudes of mothers regarding immunization.

Characteristics	Attitude		Total	Odds ratio (95% CI)*	*p-*value
	Positive (%) *n* = 105	Negative (%) *n* = 91			
Mothers’ age					
24–29	40 (53.3)	35 (46.7)	75	Ref	
30–39	58 (52.3)	53 (47.7)	111	0.95 (0.53–1.72)	0.88
40–46	7 (70.0)	3 (30.0)	10	2.04 (0.49–8.50)	0.32
Occupation					
Housewife	54 (54.5)	45 (45.5)	99	Ref	
Employee	14 (35.0)	26 (65.0)	40	0.45 (0.21–0.96)	0.03
Medical staff	17 (73.9)	6 (26.1)	23	2.36 (0.86–6.49)	0.09
Entrepreneur	8 (50.0)	8 (50.0)	16	0.83 (0.29–2.39)	0.73
Other	12 (66.7)	6 (33.3)	18	1.66 (0.58–4.79)	0.34
Mothers’ education					
Bachelor degree	79 (62.7)	47 (37.3)	126	Ref	
High school	25 (41.7)	35 (58.3)	60	0.42 (0.22–0.79)	0.007
Elementary–middle school	1 (10.0)	9 (90.0)	10	0.06 (0.008–0.53)	0.011
Number of children					
1	26 (49.1)	27 (50.9)	53	Ref	
2	65 (55.6)	52 (44.4)	117	1.29 (0.67–2.48)	0.43
3–4	14 (53.8)	12 (46.2)	26	1.21 (0.47–3.10)	0.69

⁎ Logistic regression

When mothers’ behavior towards child immunization was analyzed, most participants (93.9%) agreed with the government vaccination program and with child immunization during the COVID-19 pandemic (82.7%), but there remained some who did not attend the scheduled vaccination program (16.3%), and even higher proportions who did not recommend vaccination to others during the COVID-19 pandemic (33.7%), or who hesitated to vaccinate their children (57.7%) or attend the medical facility (48.5%) Supplementary Table 3). Mothers who were employees and those with a lower educational background were less likely to show positive behavior (Table 4).

**Table 4:** Association between demographic characteristics and behaviors of mothers regarding immunization.

Characteristics	Behavior		Total	Odds ratio (95% CI)*	*p-*value
	Positive (%) *n* = 156	Negative (%) *n* = 40			
Mothers’ age					
24–29	56 (74.7)	19 (25.3)	75	Ref	
30–39	93 (83.8)	18 (16.2)	111	1.75 (0.85–3.62)	0.13
40–46	7 (70.0)	3 (30.0)	10	0.79 (0.18–3.37)	0.75
Occupation					
Housewife	80 (80.8)	19 (19.2)	99	Ref	
Employee	25 (62.5)	15 (37.5)	40	0.39 (0.17–0.89)	0.02
Medical staff	23 (100)	0 (0.0)	23	11.38 (0.66–195.75)	0.09
Entrepreneur	13 (81.3)	3 (18.8)	16	1.03 (0.26–3.97)	0.97
Other	15 (83.3)	3 (16.7)	18	1.18 (0.31–4.52)	0.80
Mothers’ education					
Bachelor degree	115 (91.3)	11 (8.7)	126	Ref	
High school	39 (65.0)	21 (35.0)	60	0.18 (0.08–0.40)	< 0.01
Elementary–middle school	2 (20.0)	8 (80.0)	10	0.02 (0.004–0.127)	< 0.01
Number of children					
1	37 (69.8)	16 (30.2)	53	Ref	
2	98 (83.8)	19 (16.2)	117	2.23 (1.03–4.80)	0.04
3–4	21 (80.8)	5 (19.2)	26	1.81 (0.58–5.66)	0.30

⁎ Logistic regression

Mothers with a higher education level were associated with a higher child immunization rate (61%) than those with high school education (33%, *p* < 0.05) or elementary–middle school education (10%, *p* < 0.05). In contrast, mothers’ occupations, ages, and numbers of children were not associated with child immunization (Table 5). Mothers with moderate knowledge (OR 2.65, 95% CI 1.08–6.61), negative attitude (OR 5.33, 95% CI 2.71–10.59), and negative behavior (OR 7.88, 95% CI 3.36–19.47) were less likely to vaccinate their children (Table 6).

**Table 5:** Association between mothers’ sociodemographic characteristics and child immunization.

Characteristics	Immunization		Total	*p-*value*
	Incomplete (%) *n* = 73	Complete (%) *n* = 123		
Mothers’ age				
24–29	32 (42.7)	43 (57.3)	75	0.450
30–39	38 (34.2)	73 (65.8)	111	
40–46	3 (30.0)	7 (70.0)	10	
Occupation				
Housewife	38 (38.4)	61 (61.6)	99	0.139
Employee	20 (50.0)	20 (50.0)	40	
Medical staff	5 (21.7)	18 (78.3)	23	
Entrepreneur	6 (37.5)	10 (62.5)	16	
Other	4 (22.2)	14 (77.8)	18	
Mothers’ education				
Bachelor degree	49 (38.9)	77 (61.1)	126	0.001
High school	15 (25.0)	45 (75.0)	60	
Elementary–middle school	9 (90.0)	1 (10.0)	10	
Number of children				
1	18 (34.0)	35 (66.0)	53	0.846
2	45 (38.5)	72 (61.5)	117	
3–4	10 (38.5)	16 (61.5)	26	

⁎ Chi-squared

**Table 6:** The association between mother's knowledge, attitude, and behavior towards child's immunization.

Characteristics	Immunization		Total	Odds ratio (95% CI)*	*p-*value
	Incomplete (%) *n* = 73	Complete (%) *n* = 123			
Knowledge					
High	13 (24.5)	40 (75.5)	53	Ref	
Moderate	25 (46.3)	29 (53.7)	54	2.65 (1.08–6.61)	0.018
Low	35 (39.3)	54 (60.7)	89	1.99 (0.89–4.64)	0.07
Attitude					
Positive	21 (20.0)	84 (80.0)	105	Ref	0.001
Negative	52 (57.1)	39 (42.9)	91	5.33 (2.71–10.59)	
Behavior					
Positive	43 (27.6)	113 (72.4)	156	Ref	0.001
Negative	30 (75.0)	10 (25.0)	40	7.88 (3.36–19.47)	

⁎ Logistic regression

## Discussion

Our study evaluated the association between mothers’ sociodemographic characeristics, knowledge, attitude, and behavior and child immunization status in Medan, Indonesia. It showed that only a quarter of mothers had a high level of knowledge, and that higher education was associated with a higher level of knowledge, as previously reported [6,9]. There remained some misconceptions among mothers that vaccines contain nutritional supplements, are growth factors, contribute to the child's brain development, and can prevent non-communicable diseases. However, misconceptions on vaccine safety remained low, recorded in fewer than 10% of participants. Most mothers already knew that immunization does not cause autism or impotency.

The more common misconceptions among the mothers in this study were not associated with perceived negative effects of vaccines; however, the attitudes of mothers towards immunization remained split between positive and negative. Negative attitudes were usually associated with low knowledge [6,7], although in the current study attitudes were likely to be associated with the COVID-19 pandemic, with some mothers choosing neutral responses regarding compliance with vaccination schedules. A previous report in Indonesia suggested that the decision to seek immunization during the COVID-19 pandemic was highly influenced by the spouse, with only 27% deciding for themselves [5].

Our study showed that most mothers exhibited positive behavior, which was in line with a previous study [6]. The majority of them agreed with the government program on vaccination and specifically the childhood immunization program during the COVID-19 pandemic, and that children should receive vaccinations as scheduled. However, mothers were still hesitant to vaccinate their children, because they were afraid of going to the medical facility and becoming infected by the COVID-19 virus — as reported by other surveys in different parts of Indonesia [5].

The fear of contracting COVID-19 could be one of the factors that impacted immunization coverage in North Sumatera province, where the basic immunization coverage dropped to 75.5% in 2020, before increasing slightly to 80.7% in 2021 [4]. This was in line with the low basic immunization coverage (63%) among children in our study. Whether this was a result of restrictions and policies relating to travel and movement, availability of vaccine supply, or COVID-19 fears requires further evaluation. Our study did not investigate other factors, including distance/traveling time to healthcare centres, means of transport, economic status, or sources of information about infant immunization, which may have further affected mothers’ knowledge levels, attitudes, and behaviors regarding vaccination during the COVID-19 pandemic.

Control measures to curb the spread of COVID-19 in Indonesia began in March 2020. Although lockdown steps were never implemented, strong measures, including mandatory school and workplace closing, restrictions on public transportation and gathering in public places, and encouragement to stay at home and restrict movement were applied [10]. Most restrictions remained in place even until the end of our data collection in September 2021. Mothers with limited access to private transportation may not have felt safe traveling on public transportation to visit the healthcare facilities. This may have further limited access to healthcare for at least 18 months after control measures were first introduced.

The Indonesian Pediatric Society continued to the recommend routine immunization services amid the travel restriction measures. However, other factors, including staff redeployment to the COVID-19 response, PPE shortages, and non-established safety protocols in healthcare settings might have limited the availability of immunization services [2,5,11].

Previous disruptions to routine childhood immunizations due to infectious disease outbreaks, such as Ebola, have been reported [12,13]. Declines in DTP3 and MCV1 coverage have led to immunity gaps and subsequently measles outbreaks [2]. During the COVID-19 pandemic, the disruption of immunization coverage also led to increased new cases of polio in Pakistan and Afghanistan [14,15], and a new polio outbreak in Niger [16]. The health impacts of vaccine interruption have also been reported in Indonesia, where a polio case was detected in Aceh province [17].

Efforts to prevent further outbreaks due to immunization disruptions should include the acceleration of catch-up immunization and mobile immunization by outreach services in hard-to-reach areas, to target children who have missed their vaccinations [3].

## Conclusion

During the COVID-19 pandemic, the disruption of immunization services resulted in low immunization coverage. This could lead to the emergence or reemergence of vaccine-preventable diseases. Our study showed that mothers’ attitudes, behaviors, and educational backgrounds were associated with child immunization status. Recovery efforts to improve immunization coverage are urgently needed, and should include efforts to reduce mothers’ hesitancy regarding vaccination associated with the pandemic.

## Conflicts of interest

None declared

## Appendix Supplementary materials

Image, application 1
