# Supplementary material for: Mothers’ knowledge, attitude, and behavior regarding child immunization, and the association with child immunization status in Medan City during the COVID-19 pandemic
Source: IJID Reg. 2023 May 4;8(Suppl):S22–6. doi: 10.1016/j.ijregi.2023.03.014 (PMC10157388; doi:10.1016/j.ijregi.2023.03.014)
Supplement: Supplementary file 1 [file mmc1.docx]

**Supplementary Table 1**. Mother’s knowledge towards child’s immunization.

| **Knowledge domain** | **True**  **(%)** | **False**  **(%)** | **Don’t Know (%)** |
| --- | --- | --- | --- |
| Vaccine contains nutritional supplement | 71 (36.2) | 99 (50.5) | 26 (13.3) |
| Vaccine is a growth factor | 80 (40.8) | 89 (45.4) | 27 (13.8) |
| Immunization contributes in child’s brain development | 67 (34.2) | 100 (51) | 29 (14.8) |
| Immunization prevents non-communicable diseases | 86 (43.9) | 101 (51.5) | 9 (4.6) |
| Immunization can cause autism | 12 (6.1) | 145 (74) | 39 (19.9) |
| Immunization can cause impotency and should be avoided | 15 (7.7) | 135 (68.9) | 46 (23.5) |
| If a vaccine dose is missed, that vaccine should be restarted from the beginning | 34 (17.3) | 108 (55.1) | 54 (27.6) |
| If a vaccine dose is missed, visit medical facility and ask for advice | 172 (87.8) | 8 (4.1) | 16 (8.2) |

**Supplementary Table 2**. Mother’s attitude towards child’s immunization.

| **Attitude domain** | **Strongly Agree**  **(%)** | **Agree (%)** | **Neutral (%)** | **Disagree (%)** | **Strongly Disagree (%)** |
| --- | --- | --- | --- | --- | --- |
| Immunization can keep your children healthy. | 71 (36.2) | 80 (40.8) | 36 (18.4) | 5 (2.6) | 4 (2) |
| Healthy children don’t need to be vaccinated. | 6 (3.1) | 11 (5.6) | 33 (16.8) | 84 (42.9) | 62 (31.6) |
| Vaccination provides more beneficial than harmful. | 55 (28.1) | 94 (48) | 38 (19.4) | 6 (3.1) | 3 (1.5) |
| Child’s immunization in COVID-19 pandemic is important. | 65 (33.2) | 95 (48.5) | 23 (11.7) | 9 (4.6) | 4 (2) |
| Compliance to vaccination schedule in COVID-19 pandemic is important. | 63 (32.1) | 57 (29.1) | 68 (34.7) | 6 (3.1) | 2 (1) |
| Vaccination can trigger child’s immune system to fight the disease. | 75 (38.3) | 80 (40.8) | 31 (15.8) | 9 (4.6) | 1 (0.5) |
| Immunization can prevent infectious disease. | 78 (39.8) | 79 (40.3) | 33 (16.8) | 5 (2.6) | 1 (0.5) |
| Vaccinated children can still be infected by the disease. | 47 (24) | 55 (28.1) | 49 (25) | 33 (16.8) | 12 (6.1) |
| In case of emergence of vaccine preventable disease, non-vaccinated children should be quarantined as they can develop serious illness. | 36 (18.4) | 55 (28.1) | 59 (30.1) | 29 (14.8) | 17 (8.7) |

**Supplementary Table 3**. Mother’s behavior towards child’s immunization.

| **Behavior domain** | **Yes (%)** | **No (%)** |
| --- | --- | --- |
| Do you agree with government vaccination program? | 184 (93.9) | 12 (6.1) |
| Do you agree with child’s immunization in COVID-19 pandemic? | 162 (82.7) | 34 (17.3) |
| Do you attend vaccination as scheduled? | 164 (83.7) | 32 (16.3) |
| Do you recommend vaccination to others in COVID-19 pandemic? | 130 (66.3) | 66 (33.7) |
| Are your child still vaccinated when he/she is having mild illness? | 99 (50.5) | 97 (49.5) |
| Are your child still vaccinated when he/she is in serious condition? | 108 (55.1) | 88 (44.9) |
| Do you hesitate to vaccinate your child because you are afraid of getting infected by COVID-19 virus? | 83 (42.3) | 113 (57.7) |
| Do you hesitate to vaccinate your child because you are afraid of going to medical facility? | 101 (51.5) | 95 (48.5) |
